# Relationships Between Physical Activity, Sleep, Psychological Well-Being, and Academic Performance Among Native American College Students

**DOI:** 10.3390/ijerph23040491

**Published:** 2026-04-13

**Authors:** Olutosin Sanyaolu, Brandy Reeves-Doyle, Afolakemi C. Olaniyan, Tarenina Max, Adetoun Asala, Esther Osime

**Affiliations:** 1 Public Health Department, School of Arts and Sciences, Fort Lewis College, Durango, CO 81301, USA; 2 Department of Kinesiology, Nutrition, and Health, Miami University, Oxford, OH 45056, USA; reevesbn@miamioh.edu; 3 Department of Public Health, School of Population and Health Sciences, Dillard University, New Orleans, LA 70122, USA; aolaniyan@dillard.edu; 4 HIV Surveillance and Epidemiology Unit, Pennsylvania Department of Health, Harrisburg, PA 17120, USA; 5 Department of Community and Behavioral Health, East Tennessee State University, Johnson City, TN 37614, USA; osimee@mail.etsu.edu

**Keywords:** physical activity, sleep duration, psychological well-being, academic performance, Native Americans

## Abstract

**Highlights:**

**Public health relevance—How does this work relate to a public health issue?**

- Native American college students remain underrepresented in higher education and face stressors affecting well-being.
- Physical activity and sleep are modifiable health behaviors linked to psychological functioning and academic outcomes in young adult populations.

**Public health significance—Why is this work of significance to public health?**

- This study provides population-level evidence on the associations between physical activity, sleep duration, psychological well-being, and academic performance among Native American college students.
- Findings identified sleep and meaningful physical activity as actionable targets to support Indigenous student wellbeing.

**Public health implications—What are the key implications or messages for practitioners, policy makers and/or researchers in public health?**

- Institutions serving Indigenous students should integrate culturally responsive wellness initiatives that promote restorative sleep and meaningful movement.
- Future longitudinal and community-engaged research is needed to explore pathways linking health behaviors and academic success in Indigenous higher education settings.

**Abstract:**

Background: College students’ well-being is a critical determinant of academic success, and for Native American students, cultural strengths, resilience, and community support are key in fostering persistence in higher education. Alongside these assets, health behaviors are key contributors to psychological well-being (PWB) and academic performance. This study examined how modifiable health behaviors, such as physical activity (PA) and sleep duration, relate to PWB and academic performance among Native American college students. Methods: A secondary data analysis was conducted using a nationally representative sample of Native Americans (N = 1914) from the Spring 2023 American College Health Association-National College Health Assessment (ACHA-NCHA) survey. Independent variables include meeting PA guidelines (≥150 min moderate or ≥75 min vigorous/week) and sleep duration (categorized as poor or good). The Diener Flourishing Scale measured PWB. Academic performance was measured based on self-reported cumulative grade averages. Findings: Biological sex and PA were significantly associated, χ^2^ = 40.60, *p* < 0.001, with a higher proportion of males meeting PA guidelines. Students with good sleep reported higher PWB than others, *F*(1, 1817) = 62.08, *p* < 0.001. Similarly, students who met PA guidelines reported higher PWB, *F*(1, 1817) = 35.71, *p* < 0.001. Poor sleep was associated with lower odds of higher academic performance (*B* = −0.33, *p* < 0.001). Contrarily, PA was not significant (*p* = 0.350). PWB was positively associated with academic performance (*B* = 0.031, *p* < 0.001). Conclusions: Sleep and PWB are key factors associated with both PWB and academic performance, while PA is associated with PWB. These findings highlight the importance of relevant interventions that promote these factors to support overall well-being, academic success, and retention among Native American college students.

## 1. Introduction

The holistic well-being of college students, which encompasses physical, emotional, social, spiritual, and occupational wellness, has received increased attention [1,2]. College life exposes students to many challenges, which may affect their well-being and academic performance. Students often struggle with workload demands, financial pressures, social integration, and maintaining a healthy lifestyle [3]. Balancing these demands can be challenging and may lead to increased stress levels, anxiety, and a negative impact on their well-being [4]. Recent research indicates that symptoms of anxiety, depression, and psychological distress have increased substantially among university students over the past decade, highlighting a growing public health concern in higher education and an important determinant of academic persistence and success [5,6]. These experiences may be particularly unique to Native American college students, as their well-being may also be influenced by historical, cultural, and structural contexts that shape health and educational outcomes [7]. According to Indigenous public health scholarship, well-being is deeply connected to cultural identity, a sense of community belonging, and relationships with culture, land, and the Indigenous knowledge system, reflecting a holistic view of health that brings together physical, emotional, spiritual, and communal dimensions [8]. Research on Indigenous health highlights the lasting effects of historical trauma caused by colonization, forced displacement, and assimilation policies on the mental and physical health of Indigenous communities across generations. These historical and structural factors, including systemic inequities in education and limited access to culturally responsive resources, may shape Native American students’ experiences in higher education [9]. In the same vein, recent scholarship emphasizes strengths-based and culturally grounded approaches to Indigenous health that recognize resilience, cultural continuity, and community engagement as central components of well-being [10]. Despite these strengths, Indigenous students often face substantial barriers to college retention and completion [11].

Approximately 1% of the U.S. population identifies as American Indian or Alaska Native. Among those aged 25 years and older, about 16.8% have earned a bachelor’s degree or higher, compared with 36.2% of the overall U.S. population [12]. Furthermore, in fall 2024, American Indian and Alaska Native students accounted for approximately 0.6% of total postsecondary enrollment [12]. Trends over time also show continued challenges. Between Fall 2014 and Fall 2024, Native American student enrollment declined by 15.4%, from 145,900 to 123,500 students, with undergraduate enrollment decreasing by 16.9% during this period [12]. This decline raises concerns about retention and graduation among Native American college students. 

In the context of these educational disparities, it is important to assess factors that may promote Native American students’ well-being and academic success. Lifestyle factors, such as physical activity (PA) and sleep duration, may play an important role in shaping psychological well-being (PWB) [13,14,15,16]. Research posits that insufficient PA and poor sleep habits can contribute to heightened anxiety, depression, and cognitive difficulties, all of which can have a detrimental impact on academic performance [15,16]. Physical activity has well-documented benefits for psychological well-being [14,16]. Studies show that regular PA can lower symptoms of depression and anxiety, enhance mood, and help with the development of good mental health [17,18]. For college students, PA is an important mechanism for coping with academic and social stressors and building resilience [19,20,21]. Despite the benefits of PA, little is known about PA levels among Native American college students, as few studies have reported on this population. However, existing evidence from broader Native American adult populations points to higher obesity prevalence and lower PA participation compared to other racial/ethnic groups [21]. These patterns suggest that PA is a potentially important factor for Native students’ well-being, but one that remains understudied. Another study that reported on PA practices among Native Americans suggests that more than 50% of college-aged Native American adults do not meet the recommended PA guidelines of 150 min. per week of moderate-intensity or 75 min. per week of vigorous-intensity [22]. In Nez’s 2024 study, walking and dancing were more common among Native Americans who engaged in PA [22]. Traditional forms of PA, such as dancing, also offer Native American students a chance to preserve their culture, stay physically fit, enhance spiritual well-being, and foster a sense of community and identity [23]. The findings highlight the importance of culturally tailored PA interventions, particularly those incorporating traditional activities like dance, to improve PA levels and mitigate health disparities among Native American college students. These interventions would provide students with an opportunity to preserve their cultural heritage and incorporate it into their identity, thereby enhancing their PWB [24]. 

Sleep duration is another predictor of PWB [25]. Research has documented associations between poor sleep and adverse mental health outcomes, including depression, anxiety, and decreased cognitive functioning [26]. Erratic sleep patterns and insufficient sleep are common among college students, impeding academic performance and PWB [27,28]. Evidence suggests that sleep duration, quality, and regularity are positively associated with academic outcomes and cognitive performance among university students [29,30]. However, findings are not entirely consistent. For example, Jalali et al. (2020) reported mixed results, with no consistent association between sleep quality and academic performance [31]. Although several studies have examined sleep across different racial and ethnic groups, there is a paucity of information regarding Native Americans [32]. Previous research suggests that Native American adults are less likely to achieve healthy sleep of more than seven hours compared to other racial and ethnic groups [32]. The paucity of data on Native American college students’ sleep patterns and their associated impacts underscores the need for more comprehensive research to understand and address the unique challenges faced by this population. In this context, it is also essential to assess gender differences, as females may experience elevated risks. Research indicates that females report poor sleep patterns and are more likely to have sleep problems than males [33]. These gender-based differences in sleep could have an impact on psychological well-being, and examining whether this pattern is similar among Native American students would help determine the need for gender-specific strategies to address sleep problems in this population.

Physical activity and sleep are highly interrelated and bidirectional [34]. Research has shown that higher levels of PA can lead to improved sleep, and that better sleep can, in turn, contribute to increased PA [34,35]. These relationships align with the biopsychosocial model of health, which posits that health outcomes are shaped by interactions among biological, psychological, and social factors [36]. Within this framework, lifestyle behaviors such as PA and sleep influence psychological well-being, which may in turn affect academic success. Research has shown that psychological issues and physical health issues are associated with higher college dropout rates and lower success rates [5,13]. Recognizing that students’ well-being is a key determinant of persistence in higher education, our focus is to identify modifiable behaviors that may strengthen Native American students’ success in college. Understanding these relationships is particularly important for Native American college students, whose well-being may also be influenced by broader contextual factors such as cultural identity, community belonging, and social support. This perspective is also consistent with Indigenous views of health that emphasize holistic wellness and the interconnected roles of physical health behaviors, psychological well-being, cultural identity, and community relationships in shaping overall health outcomes among Native American populations [37,38]. Therefore, this study aimed to examine the relationships between PA and sleep duration and their associations with PWB and academic performance among Native American college students. Additionally, we explored potential gender differences across these variables.

## 2. Method

### 2.1. Participants and Procedure

This study conducted a secondary analysis of the spring 2023 American College Health Association-National College Health Assessment (ACHA-NCHA) data. The ACHA-NCHA is a nationally recognized survey designed to help college health service providers, health educators, counselors, and administrators collect data on students’ habits and behaviors related to the most prevalent health topics. The survey assists in collecting precise data on a wide range of health and wellness issues that impact the student population and their academic performance [39]. The Spring 2023 web-based survey included 78,024 respondents aged 18 years and above from 125 participating colleges and universities across the United States. Institutions opt to participate in the survey, and interested institutions send it to their students via email. Participation was confidential, and the response rate was 11.0%. Of the 78,024 college students who participated in the survey, 1914 (2.5%) students who self-identified as Native Americans (American Indians or Native Alaskans) were included in this study.

### 2.2. Measures

#### 2.2.1. Independent Variables: Physical Activity

Physical activity level was assessed based on items from the survey, which asked respondents to report the amount of moderate and vigorous intensity PA performed the previous week. Responses were used to determine whether participants met the PA Guidelines for Americans, defined as engaging in ≥150 min of moderate-intensity aerobic activity per week, or ≥75 min of vigorous-intensity activity [40]. Based on these guidelines, the NCHA created a binary variable, PAGUIDE, that classified students as meeting or not meeting the recommended PA guidelines. PAGUIDE, categorized as ‘Yes’ or ‘No’, was used to assess PA levels in this study.

#### 2.2.2. Independent Variables: Sleep Duration

This study assessed sleep duration using variable N3Q14: “Over the last 2 weeks, what is the average amount of sleep you have gotten on a weeknight (excluding naps)?” Response options were less than 4 h, 4 h, 5 h, 6 h, 7 h, 8 h, 9 h, or 10 h or more.

Sleep duration was categorized based on the National Sleep Foundation’s recommended sleep duration of 7–9 h for young adults and adults [41]. Those who reported fewer than 7 h of sleep were categorized as having poor sleep duration, and those who reported at least 7 h of sleep were categorized as having good sleep duration.

#### 2.2.3. Dependent Variable: Psychological Well-Being

PWB was assessed using the Flourishing scale developed by Diener et al., 2010 [42]. This is an eight-item measure of respondents’ self-perceived success in important areas, including relationships, competence, purpose and meaning in life, engagement in daily activities, optimism, and contribution to others’ well-being. Each item is rated on a 7-point Likert scale ranging from strongly disagree (1) to strongly agree (7). PWB scores ranged from 8 to 56, with higher scores indicating a higher PWB. The scale has demonstrated good internal consistency reliability, with a reported Cronbach’s alpha of 0.87 [42]. 

#### 2.2.4. Dependent Variable: Academic Performance

This study assessed academic performance based on self-reported approximate cumulative grade averages. Those with a cumulative grade average of A were coded as 4, B as 3, C as 2, D as 1, and F as 0.

#### 2.2.5. Covariates

The following variables were included as covariates to control for potential confounding effects:

- Biological sex (male, female)
- Age (18–24 years, 25 years and above)
- Academic year
  - Undergraduate, Lower Division: 1st and 2nd year
  - Undergraduate, Upper Division: 3rd to 5th year
  - Graduate: master’s or doctoral level

### 2.3. Data Analysis

Data analysis was conducted using Statistical Package for Social Sciences (SPSS), version 27.0. This study utilized various statistical tests to examine the relationships between PA, sleep duration, PWB, and academic performance. First, we analyzed descriptive statistics to summarize the key variables and conducted independent-samples *t*-tests to compare mean PWB scores by biological sex. To test differences in PWB by PA and sleep duration, a univariate General Linear Model (GLM) was employed with PA and sleep as fixed factors, adjusting for covariates including biological sex, age, and academic year. We also tested for an interaction effect between PA and sleep on PWB.

To test differences in PWB by PA and sleep duration, a univariate GLM was employed with PA and sleep as fixed factors, adjusting for covariates including biological sex, age, and academic year. We also tested for an interaction effect between PA and sleep on PWB.

In addition, Chi-square tests were conducted to investigate the association between biological sex, PA, sleep duration, and academic performance. For academic performance, because the outcome was ordinal, a Mann–Whitney U test was used to examine differences between male and female students.

An ordinal logistic regression (proportional odds model) was conducted to examine whether PA, sleep duration, PWB, and demographic variables (biological sex, age category, and academic year) predicted academic performance. Moderation analyses were also conducted to examine whether PWB moderates the associations of sleep duration and PA with academic performance. Ordinal logistic regression models were estimated for sleep duration and PA, with interaction terms entered for sleep duration*PWB and PA*PWB, respectively. Prior to analysis, missing data were handled using listwise deletion. Only cases with complete data on variables included in each analysis were retained. 

Model assumptions were assessed prior to analysis. For the GLM, homogeneity of variance was evaluated using Levene’s test. For the ordinal regression models, the proportional odds (parallel lines) assumption was assessed. Multicollinearity among predictors was examined using variance inflation factors (VIF), with all VIF values ranging from 1.00 to 2.00, indicating no evidence of multicollinearity. Effect sizes were reported using partial eta squared (η^2^) for GLM analyses and Nagelkerke R^2^ for ordinal regression models.

## 3. Results

The sample included 1914 Native American college students, with the majority aged 18–24 (64.8%). Participants included students from across levels of study: 42.8% were in the lower division (1st- and 2nd-year undergraduate students), 40.3% were in the upper division (3rd- to 5th-year undergraduate students), and 16.9% were graduate students. Regarding GPA, 86.5% of participants reported a CGPA of at least a “B”. Approximately 72% of the participants were female, while 28.1% were male. The mean Diener Flourishing score (PWB) was 44.26. Approximately 45.0% of respondents met the recommended guidelines for aerobic activity, while about 50.8% reported sleeping an average of 7 h or more. See Table 1.

### 3.1. Physical Activity, Sleep Duration, and Psychological Well-Being

We examined differences in PWB among Native American college students using a univariate general linear model with sleep duration and PA as fixed factors while adjusting for biological sex, age, and academic year. Levene’s test was significant, *F*(47, 1777) = 2.21, *p* < 0.001, indicating unequal variances across groups; however, the analysis was considered robust given the large sample size. The overall model was significant, *F*(7, 1817) = 18.55, *p* < 0.001, partial η^2^ = 0.067. 

Students with good sleep reported higher PWB than those with poor sleep, *F*(1, 1817) = 62.08, *p* < 0.001, partial η^2^ = 0.033, with an adjusted mean difference of 2.97, 95% CI: 1.69−4.24 (good sleep: *M* = 45.19, SE = 0.30; poor sleep: *M* = 41.33, SE = 0.30). Similarly, students who met PA guidelines reported higher PWB than those who did not, *F*(1, 1817) = 35.71, *p* < 0.001, partial η^2^ = 0.019, with an adjusted mean difference of 2.18 points, 95% CI: 0.97–3.39.

Females reported slightly higher PWB than males, F(1, 1817) = 3.98, *p* = 0.046, partial η^2^ = 0.002, with an adjusted mean difference of 0.98 points, 95% CI: 0.02–1.94, (female: *M* = 44.53, SE = 0.25; male: *M* = 43.89, SE = 0.44). Older students (25 years and above) reported significantly higher PWB than younger students (18–24 years), *F*(1, 1817) = 21.28, *p* < 0.001, partial η^2^ = 0.012, with an adjusted mean difference of 2.35 points, 95% CI: 1.35–3.35 (25 years and above: *M* = 45.73, SE = 0.39; 18–24 years: *M* = 43.63, SE = 0.27). However, academic year was not a significant predictor of PWB, *F*(2, 1817) = 0.47, *p* = 0.627, partial η^2^ = 0.001. The interaction between sleep and PA was also not significant, *F*(1, 1817) = 1.23, *p* = 0.267, partial η^2^ = 0.001. This suggests that PA and good sleep duration independently contribute to higher PWB, with no added interactive effect between the two variables. 

### 3.2. Biological Sex, Physical Activity, and Sleep Duration

We compared biological sex with meeting the weekly PA guidelines and sleep duration among Native American college students using Chi-squared test. The results indicated a significant association between biological sex and PA, χ^2^ (1, N = 1902) = 40.60, *p* < 0.001. More males than females met the weekly PA guideline. Specifically, 56.72% of males met the guideline, compared to 40.55% of females.

Another Chi-square test was performed to examine the relationship between biological sex and sleep duration. The results showed no statistically significant association between biological sex and sleep duration, χ^2^ (1, N = 1898) = 0.36, *p* = 0.550. Approximately 49.0% of females reported poor sleep duration, while 51.4% reported good sleep duration. Among males, 50.1% reported poor sleep duration, while 49.9% reported good sleep duration. This suggests that males and females in the sample reported similar sleep patterns, with nearly equal proportions achieving less than seven hours of sleep.

### 3.3. Biological Sex and Psychological Well-Being

An independent samples t-test was used to examine differences in PWB between male and female participants. Levene’s test for equality of variances was significant, *F*(1, 1877) = 14.18, *p* < 0.001. This indicates a violation of the assumption of homogeneity. Therefore, results from the unequal variances (Welch’s t-test) were interpreted. The results indicated that there was no significant difference in PWB scores between females (*M* = 44.50, SD = 9.08) and males (*M* = 43.80, SD = 10.88), t(833.63) = −1.32, *p* = 0.188, 95%CI: −1.75–0.34 The small effect size, d = −0.07, 95% CI: −0.17–0.03, indicates a negligible difference in PWB between males and females.

### 3.4. Biological Sex and Academic Performance

A Mann–Whitney U test was conducted to examine the differences in academic performance between male and female students. The results showed no significant difference in academic performance between females (Median = 4, Mean Rank = 959.50) and males (Median = 4, Mean Rank = 920.52), U = 349,096.5, Z = −1.54, *p* = 0.124. Although females had a higher mean rank, the difference was not statistically significant.

### 3.5. Relationship Between Physical Activity, Sleep Duration, and Academic Performance

We examined whether sleep duration, PA, participants’ age, biological sex, and academic year predicted academic performance using ordinal logistic regression. The final model was statistically significant, χ^2^(6) = 113.62, *p* < 0.001. Model fit indices suggested a good fit to the data, as both the Pearson χ^2^ (182) = 193.24, *p* = 0.270 and Deviance χ^2^(182) = 162.996, *p* = 0.841 tests were non-significant. No evidence of multicollinearity was observed among predictors, and the proportional odds (parallel lines) assumption was not violated (χ^2^(18) = 28.28, *p* = 0.058). The model explained a modest proportion of variance in academic performance (Nagelkerke R^2^ = 0.068).

Among the predictors, sleep duration and academic year significantly predict academic performance. Students who reported poor sleep had lower odds of reporting higher academic performance compared to those with good sleep (B = −0.33, SE = 0.09, Wald = 12.80, *p* < 0.001, 95% CI: −0.50–−0.15). Compared to graduate students, lower division students had significantly lower odds of higher academic performance (B = −1.33, SE = 0.16, Wald = 70.31, *p* < 0.001, 95% CI:−1.64–−1.02), as did upper division students (B = −1.14, SE = 0.16, Wald = 54.49, *p* < 0.001, 95% CI: −1.45–−0.84). In contrast, PA was not a significant predictor (B = −0.09, SE = 0.09, Wald = 0.87, *p* = 0.350, 95% CI: −0.27–0.10). Similarly, age category was not significantly associated with academic performance (B = −0.04, SE = 0.11, Wald = 0.11, *p* = 0.738, 95% CI: −0.24–0.17). Biological sex was also not a statistically significant predictor (B = −0.18, SE = 0.10, Wald = 3.22, *p* = 0.073, 95% CI: −0.38–0.02).

A moderation analysis was conducted to assess whether PWB moderates the effects of sleep duration and PA on academic performance (GPA), while controlling for age, academic year, and biological sex. The overall model was statistically significant, χ^2^(7) = 147.97, *p* < 0.001. The model explained a modest proportion of variance in academic performance (Nagelkerke R^2^ = 0.088). PWB was a significant predictor of academic performance. Higher PWB was associated with increased odds of reporting higher academic performance (B = 0.031, SE = 0.006, Wald = 25.98, *p* < 0.001, 95% CI: 0.019–0.043). Sleep duration was also a significant predictor. Students with poor sleep had lower odds of reporting higher academic performance compared to those with good sleep (B = −0.25, SE = 0.09, Wald = 7.25, *p* = 0.007, 95% CI: −0.43–−0.07). However, the interaction between sleep duration and PWB was not statistically significant (B = −0.007, SE = 0.010, Wald = 0.54, *p* = 0.462, 95% CI: −0.026–0.012). This indicates that PWB did not moderate the relationship between sleep duration and academic performance.

Finally, we examined whether PWB moderated the relationship between PA and academic performance, while controlling for age, academic year, and biological sex. The overall model was statistically significant, χ^2^(7) = 140.68, *p* < 0.001. The model explained a modest proportion of variance in academic performance (Nagelkerke R^2^ = 0.084). PWB was a significant predictor of academic performance, such that higher PWB was associated with increased odds of reporting higher academic performance (B = 0.029, SE = 0.006, Wald = 22.94, *p* < 0.001, 95% CI: 0.017–0.041). In contrast, PA was not a significant predictor of academic performance (B = −0.02, SE = 0.10, Wald = 0.05, *p* = 0.830, 95% CI: −0.21–0.17). The interaction between PA and PWB was also not statistically significant (B = 0.003, SE = 0.010, Wald = 0.13, *p* = 0.722, 95% CI:−0.015–0.022). This indicates that PWB did not moderate the relationship between PA and academic performance.

## 4. Discussion

This study examined the associations between PA, sleep duration, PWB, and academic performance among Native American college students in the United States. These findings offer a profound understanding of the relationships between PA and sleep duration to overall well-being and academic success among Native American students and help elucidate key variables for consideration in efforts to enhance student outcomes in higher education.

In this study, participants reported low PA levels, with more than half not meeting the recommended PA guidelines. Findings from this study support earlier studies indicating that regular PA is associated with higher PWB levels [16,43]. More specifically, the participants in our study who engaged in PA had higher Diener Flourishing Scores, suggesting a positive association between PA and PWB. This finding indicates that PA may be an important factor related to mental health and resilience against academic and social burdens among Native American college students. To promote PA, institutions could encourage engagement in outdoor activities such as games, hiking, and swimming. These activities may also be culturally adapted to resonate with students’ backgrounds. As Nez (2024) noted, traditional activities such as walking and dancing not only help Native American students maintain PA but also foster a connection to the culture [22]. Cultural relevance may enhance the psychological benefits associated with PA, suggesting that interventions incorporating traditional practices may be valuable for Native American communities [22]. Previous research has also shown that culturally tailored PA programs are associated with improved mental health outcomes by promoting a sense of identity and community [44].

Sleep duration was also significantly associated with PWB. The scores on the Diener Flourishing Scale were significantly higher among students with good sleep duration than among those with poor sleep duration. This finding aligns with previous research linking inadequate sleep with adverse mental health outcomes. Studies have also reported that insufficient sleep is associated with impaired cognitive functioning, further underscoring the importance of good sleep for PWB [26,28]. Poor sleep is a common problem among college students, and these findings highlight the importance of interventions and proper sleep hygiene, especially for Native American students who may face specific challenges in achieving enough sleep.

This study found that PA and sleep duration were independently associated with higher PWB; however, no significant interaction between them was observed. Although prior research suggests that PA and sleep are bidirectionally related, our findings indicate that associations with PWB appear to operate independently in this sample [34,35]. The findings thus indicate the importance of considering both PA and adequate sleep when developing strategies aimed at supporting PWB among Indigenous university students. 

The study also found a significant difference in levels of engagement in PA for biological sex, with a higher proportion of male students meeting the recommended PA guidelines compared with female students. This aligns with previous literature that showed that males are more likely to engage in PA than females [24]. These differences suggest that gender-specific approaches may be useful when designing programs to improve PA among Native American college students, especially for female students. However, this pattern did not extend to sleep duration. No significant association was observed between biological sex and sleep duration, indicating that male and female students exhibited similar sleeping patterns. This is consistent with previous research suggesting no gender differences in sleep duration for college students [33]. 

These findings highlight the importance of addressing biological sex-related differences in modifiable lifestyle factors, PA, within the college environment. At the institutional level, universities should consider embedding wellness into their academic culture by investing in facilities, peer-support systems, and education campaigns that highlight the importance of PA as a modifiable factor linked to resilience and well-being.

No significant difference in academic performance was observed between male and female students. Although females had a higher mean rank in academic performance, the difference was not statistically significant, suggesting that biological sex was not associated with academic performance in this sample. This finding corroborates broader trends in the literature, which showed no significant influence of gender on academic performance [45,46]. Although males and females may differ in health behaviors, such as PA, academic success may also be influenced by other factors not considered in this study. Future studies could incorporate additional variables, such as stress, mental health status, study environment, and social support, to better explain academic outcomes.

The relationship between PA, sleep duration, and academic performance has important implications for student retention [47]. Previous studies have reported that poor sleep and low PA levels are associated with poorer cognitive functioning, stress management, and academic outcomes [14,28]. However, findings remain mixed; for example, Jalali et al. (2020) reported no direct association between sleep and academic performance [31]. In this study, sleep duration and academic year significantly predicted academic performance, whereas PA did not. Also, PWB was an independent predictor of academic performance but did not moderate the association between sleep, PA, and academic outcomes. Although the regression model was statistically significant, the explained variance in academic performance was modest. This indicates that PA, sleep duration, and the included demographic variables account for only a limited proportion of the variation in academic outcomes. Academic performance may be influenced by broader social and contextual factors, such as stress, mental health status, financial pressures, campus belonging, and academic support systems, not included in the study.

Sleep duration and PWB each play essential roles in students’ academic success, and improving either factor may benefit students regardless of the level of the other. Promoting healthy sleep habits and PWB may therefore be important considerations when developing strategies aimed at supporting student success and retention, particularly among Native American students, who remain underrepresented in higher education [12].

These findings can also be interpreted within the biopsychosocial framework referenced in the introduction, which suggests that health outcomes emerge from interactions among biological, psychological, and social factors [36]. Indigenous perspectives on health similarly emphasize a holistic understanding of wellness that integrates physical, mental, emotional, spiritual, and community dimensions of health [38]. In this context, health behaviors such as PA and sleep may contribute to well-being not only through physiological pathways but also by strengthening cultural identity, social connection, and community belonging. Culturally responsive health promotion approaches that incorporate traditional practices, peer support, and community engagement may therefore strengthen resilience and psychological well-being among Native American college students [37,38,48]. Previous research among Indigenous populations also highlights resilience, social support, and community connectedness as important protective factors for health and well-being [49].

### 4.1. Limitations

This study is not without limitations. We conducted a secondary data analysis of a cross-sectional survey, which limits our ability to control potential confounding variables that may influence the relationships between PA, sleep duration, and PWB. Additionally, the survey relied on self-reporting, which may be susceptible to certain biases, such as social desirability bias or recall bias. For instance, participants may have inaccurately recalled the frequency or duration of their PA. Academic performance was also measured using self-reported GPA, which may not accurately reflect students’ official academic records and could be influenced by reporting errors. Sleep was measured using self-reported sleep duration, which did not capture other dimensions of sleep quality, such as sleep latency, disturbances, and daytime dysfunction. Further research may focus on longitudinal study designs and objective measures of PA and sleep to provide comprehensive evidence on these factors. This study did not account for other non-behavioral factors, such as socioeconomic status, financial hardships, or campus belonging, which may interact with sleep, PA, and PWB to influence academic performance. Future research should incorporate structural and psychosocial determinants to better understand how intersecting factors shape academic success among Native American college students.

### 4.2. Implication

This study highlights the public health relevance of promoting PA and sleep among Native American college students as strategies to enhance their psychological well-being and academic success. Institutions enrolling Native American students should develop wellness initiatives that incorporate culturally relevant physical activities to foster engagement and resilience. For example, institutions could partner with tribal leaders or cultural organizations to offer activities such as traditional dance programs, land-based physical activities, or culturally rooted wellness workshops that combine physical movement with cultural identity and community connection [7,8,9]. Sleep education programs can also address widespread barriers to adequate rest, including coursework and social obligations. Institutions may launch campus-wide sleep health campaigns, provide workshops on sleep hygiene and time management, and integrate sleep education into student orientation or wellness curricula [50]. Campus policies that support PA and sleep-conducive environments, such as flexible course scheduling and designated wellness spaces, can help strengthen student health and academic outcomes. Such approaches are crucial for improving retention and graduation rates among Native American students, who remain underrepresented in higher education. Addressing these modifiable behaviors through culturally tailored and systemic strategies can enable public health practitioners and educators to promote well-being in higher education.

## 5. Conclusions

This study contributes to the limited literature examining health behaviors and well-being among Native American college students. Both PA and adequate sleep were independently associated with higher levels of PWB, with sleep duration also demonstrating relevance for academic performance. These findings underscore the importance of promoting healthy sleep and culturally meaningful PA to strengthen student well-being. Institutional efforts should embed wellness promotion within culturally responsive and structurally supportive frameworks that acknowledge the broader contexts shaping Indigenous student experiences. Future longitudinal and community-engaged research is needed to clarify the pathways linking health behaviors, well-being, and academic outcomes in Indigenous higher education settings.

## Figures and Tables

**Table 1:** Sociodemographic and behavioral characteristics of participants (N = 1914).

Variable	n (%)
Age (n = 1894)	
18−24 years	1227 (64.8%)
≥25 years	667 (35.3%)
Biological Sex (n = 1902)	
Male	536 (28.2%)
Female	1366 (71.8%)
Academic Year (n = 1879)	
Lower division (1st−2nd year)	804 (42.8%)
Upper division (3rd−5th year)	757 (40.3%)
Graduate	318 (16.9%)
Self-reported Academic Performance (CGPA) (n = 1906)	
A	972 (51.0%)
B	677 (35.5%)
C	199 (10.4%)
D	15 (0.8%)
F	43 (2.3%)
Physical Activity	
Met PA guideline (≥150 min/week)	861 (45.0%)
Did not meet PA guideline (<150 min/week)	1053 (55.0%)
Sleep duration (n = 1914)	
≥7 h (good sleep)	971 (50.8%)
<7 h (poor sleep)	939 (49.2%)
Psychological Well-Being (PWB), mean ± SD	M = 44.26 ± 9.65

Note: PA = physical activity; PWB = psychological well-being. PA guideline defined as ≥150 min/week of moderate-intensity or ≥75 min/week of vigorous-intensity physical activity.

## Data Availability

The data presented in this study are derived from the American College Health Association National College Health Assessment (ACHA-NCHA) dataset. These data are not publicly available but may be accessed for independent analysis upon submission of a proposal to the American College Health Association.
